
JOURNAL INFORMATION
==============================
NLM Title Abbreviation: ScientificWorldJournal
Journal ID: ScientificWorldJournal
NLM Journal ID: 101131163
Journal ID: TSWJ

The Scientific World Journal

ISSN: 2356-6140
EISSN: 1537-744X
Publisher: Wiley

ARTICLE INFORMATION
==============================
PMCID: PMC5956459
PMID: 15349508
DOI: 10.1100/tsw.2004.120
Article ID: 196768
Article version: 1
Subjects: Case Study

Buried Rubber Band: A Cause of Persistent Penile Discharge, Recurrent Urinary Tract Infections, and Development of Urethrocutaneous Fistula

Karim Amer *
Institute of Urology, London
*Amer Karim: amerkarim@e320.freeserve.co.ukAcademic Editor: Anthony Atala

Publication date: 2004
Electronic publication date: 2004 Aug 13
Volume: 4
First page: 655
Last page: 656
Received 2004 May 2; Revised 2004 Aug 3; Accepted 2004 Aug 13
Copyright: Copyright © 2004 Amer Karim.
Copyright year: 2004
License: This is an open access article distributed under the Creative Commons Attribution License, which permits unrestricted use, distribution, and reproduction in any medium, provided the original work is properly cited.
License URL: https://creativecommons.org/licenses/by/3.0/

Keywords: urethral discharge, penile incarceration, fistula, urinary tract infection

==============================
Case Study
*TheScientificWorldJOURNAL* (2004) 4, 655–656
ISSN 1537-744X; DOI 10.1100/tsw.2004.120

### Buried Rubber Band: A Cause of Persistent Penile Discharge, Recurrent Urinary Tract Infections, and Development of Urethrocutaneous Fistula

Amer Karim*
*Senior House Surgeon, Institute of Urology, London*

**E-mail**: amerkarim@e320.freeserve.co.uk

*Received May 2, 2004; Accepted August 3, 2004; Published August 13, 2004*

**KEYWORDS**: urethral discharge, penile incarceration, fistula, urinary tract infection

**DOMAINS**: urology

### CASE REPORT

An 88-year-old man with no previous urological history was referred with profuse offensive urethral discharge. He gave a 2-year history of recurrent urinary tract infections and increasing difficulty and pain retracting his foreskin.

On examination, he was noted to have a very tight, erythematous painful foreskin. Urinalysis was normal and previous penile swabs and urine cultures had grown no organisms. He was resultantly counseled and listed for a routine circumcision.

At operation, the foreskin was retracted and during the dissection process, a tight rubber band (Fig. 1) was discovered buried within the mucosa (Fig. 2), under newly epithelialized skin. It was found to completely encircle the glans penis, creating a band of infected ulcerated tissue that had resulted in the formation of a small urethrocutaneous fistula. The elastic band was cut and removed and the defect was closed with absorbable sutures.

Following the operation, the patient denied any history of erectile dysfunction and did not recall ever placing the rubber band around his penis. He went on to make a full recovery and was subsequently discharged.

### COMMENTS

Penile incarceration should be considered a surgical emergency, as early decompression is associated with fewer complications secondary to penile devascularization, urinary retention, and urethral damage[1]. Solid rings are most commonly implicated, but hidden foreign bodies are also a rare cause of strangulation and urethrocutaneous fistula.

*Corresponding address: 17 Rampart Walk, Dorchester, Dorset DT1 2QZ; Tel: +44 1305266415/Fax: +44 1305266415
©2004 with author.

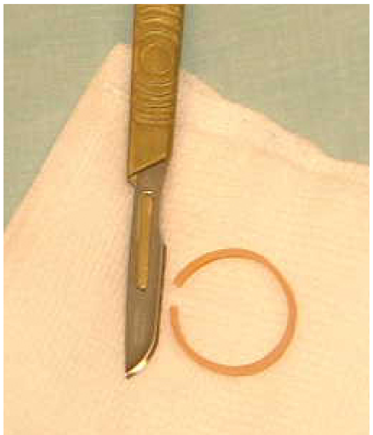

**FIGURE 1**

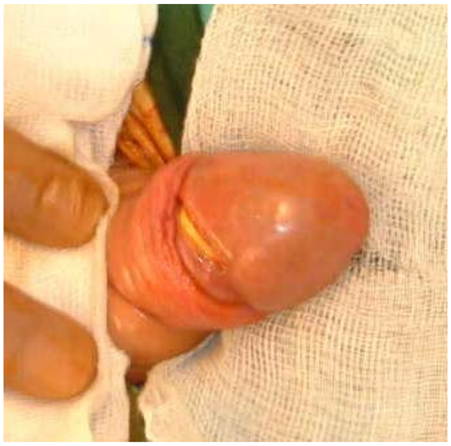

**FIGURE 2**

### REFERENCE

1. Du, J.N. (1984) Foreign-body implants in the foreskin. *Can. Med. Assoc. J.* **130(7),** 845–846.

---

**This article should be referenced as follows:**

Karim, A. (2004) Buried rubber band: a cause of persistent penile discharge, recurrent urinary tract infections, and development of urethrocutaneous fistula. *TheScientificWorldJOURNAL* **4,** 655–656.

**Handling Editor:**

Anthony Atala, Principal Editor for *Urology* — a domain of *TheScientificWorldJOURNA*L.

---
